# Optimizing Planting Density and Nitrogen Application Enhances Root Lodging Resistance and Yield via Improved Post-Anthesis Light Distribution in Sweet Corn

**DOI:** 10.3390/plants15020200

**Published:** 2026-01-08

**Authors:** Hailong Chang, Hongrong Chen, Jianqiang Wang, Qingdan Wu, Bangliang Deng, Yuanxia Qin, Shaojiang Chen, Qinggan Liang

**Affiliations:** 1 College of Agronomy and Biotechnology, China Agricultural University, Haidian District, Beijing 100193, China; hl2004@126.com (H.C.);; 2 Hainan Institute of Industrial Technology, Guangdong Academy of Science, Sanya 572024, China; 3 Institute of Nanfan & Seed Industry, Guangdong Academy of Sciences, Guangzhou 510316, China; 4 School of Breeding and Multiplication (Sanya Institute of Breeding and Multiplication), Hainan University, Sanya 572025, China

**Keywords:** sweet corn, light distribution, photosynthetic capacity, lodging resistance, yield, nitrogen application, planting density

## Abstract

Context: Optimizing nitrogen application and planting density is critical for achieving high yields and increasing lodging resistance in crops. However, the agronomic mechanisms underlying these benefits remain unclear. Objectives: This study aimed to elucidate the relationships among light distribution within the canopy, photosynthetic capacity, root architecture, yield, and lodging resistance in sweet corn. Methods: A two-year field experiment (2024–2025) was conducted using a split-plot design with two factors: nitrogen application levels as main plots (namely, N150 and N200; 150 kg/ha and 200 kg/ha, respectively) and three planting densities as sub-plots (D20, D25, and D30, representing plant spacing of 20 cm, 25 cm, and 30 cm, respectively, with a fixed row spacing of 80 cm). Results: At a given planting density, N150-treated plants exhibited significantly enhanced basal stem node strength and root architecture compared to those treated with N200. These improvements were closely associated with the increase in light interception rate (IR) into the lower canopy under N150. Consequently, root-lodging resistance increased, reducing the root lodging rate by 80.82% (7.32% vs. 13.21% under N200). Due to these advantages, the average yield of N150-treated plants was higher than that of N200-treated plants (+3.16%). Notably, increasing planting density emerged as the primary factor driving ear yield improvement, with the highest yield observed under the N150D20 group plants, which can reach ~29 t/ha. Conclusion: Coordinating nitrogen input with appropriate planting density improves vertical light distribution, particularly in the middle and lower canopy, thereby strengthening the basal stem and root systems and enhancing root lodging resistance and yield. Implication: These findings offer practical guidance for achieving high sweet corn yields by integrating canopy light management with optimized nitrogen application and planting density, and provide scientific guidance on “smart canopy” selection for sweet corn breeding.

## 1. Introduction

Sweet corn, a fresh-use variety of maize, is cultivated globally due to its high nutritional value [1]. China is the largest producer of sweet corn, but the planting density in the country is lower than in developed nations like the USA, which limits its yield potential [2]. Increasing planting density is an effective method to boost grain yield in cereal crops, which is crucial for global food security [3]. However, higher planting density can lead to plant lodging, ultimately causing yield loss [4,5]. Stem lodging occurs when stalks break at or below the ear-bearing node, severely disrupting the transport of water and nutrients between the root and above-ground parts [6,7]. The complex structural carbohydrates (such as cellulose, hemicellulose, and lignin) and the stem’s mechanical strength, particularly bending and compressive strength, have a substantial impact on lodging resistance [8,9]. The root system architecture and root differentiation (brace root and crown root development) are also key determinants of lodging resistance [10]. Balancing the root–shoot interaction is crucial to improving lodging resistance, which relies on the photosynthetic performance of the “source” organs, either at the group or individual plant level [11].

Plant architecture influences the distribution of light across canopy layers [12]. The development of leaves in the middle canopy layer is hypothesized to affect the strength of basal stem nodes, especially during the later growth stages as lower leaves senesce [6]. Weaker light conditions in the lower canopy layers of densely planted crops are thought to reduce maize stem strength, increasing the likelihood of lodging [13]. However, empirical evidence for this is limited. Canopy temperature, humidity, and photosynthetic traits of the ear leaf, such as chlorophyll content (SPAD), nitrogen content, and leaf area index, significantly influence individual plant photosynthetic capacity [14]. These parameters are heavily influenced by group plant architecture, indicating that a suitable canopy architecture can enhance photosynthetic capacity by optimizing the canopy microclimate and light distribution.

Nitrogen, a vital nutrient for crop development, plays a key role in chlorophyll and protein synthesis, promotes cell division and growth, and influences enzyme activity and nucleic acid metabolism [15]. Over recent decades, nitrogen management has contributed to yield increases in global crop production, significantly impacting food security [16]. However, excessive nitrogen use can lead to environmental pollution and adversely affect both yield and the nutritional quality of crops [17]. Efficient nitrogen management is crucial for improving nitrogen use efficiency. Our previous study demonstrated that reducing nitrogen application by 20% under dense planting conditions can improve sweet corn root architecture and trigger brace root development, which balances root absorption and anchorage capacity [6]. The split nitrogen application strategy, which adapts to crop, soil, and climate conditions, is a flexible approach to fertilization. However, it may increase the risk of root lodging due to limited brace root development, particularly when heavy summer rains occur (unpublished data).

The post-anthesis period is a critical growth stage for “sink” development in cereal crops. However, this stage is also when plant lodging often occurs [18]. Therefore, balancing the relationship between “vegetative” and “reproductive” development is key to increasing yield potential [19]. While plant architecture (light distribution) and photosynthetic capacity at the individual plant level, as well as their relationship to plant lodging in response to planting density and nitrogen application rate, have been well studied in other crops, they remain underexplored in sweet corn.

We hypothesize that optimizing planting density and nitrogen application rates can shape a favorable plant architecture that improves light distribution through the canopy layers, thereby enhancing individual plant photosynthetic capacity. This, in turn, will strengthen the development of the root system and the stem node, balance the root–shoot interaction, and ultimately improve lodging resistance. The objectives of this study are the following: (1) to investigate light distribution through canopy layers and the photosynthetic capacity of individual plants, (2) to examine the relationship between root–shoot development and light distribution, and (3) to evaluate the relationship between plant lodging and yield potential.

## 2. Results

### 2.1. Light Distribution

**Figure 1:**
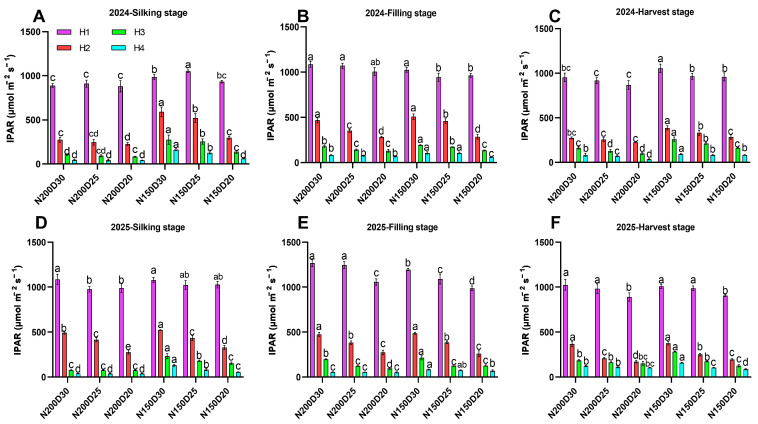
Effect of planting density and nitrogen application rates on photosynthetically active radiation (PAR) distribution across canopy layers during the post-anthesis stage in 2024 and 2025: (**A**–**C**) 2024 silking–harvest stages; (**D**–**F**) 2025 silking–harvest stages. Different lowercase letters indicate significant differences at *p* < 0.05. Error bars represent SD.

A similar trend was observed during the post-anthesis period in both years (Figure 1). Interception photosynthetically active radiation (IPAR) significantly decreased with increasing planting density under the same nitrogen application rate (*p* < 0.05). The average IPAR at H1, H2, H3, and H4 vertical height were significantly increased in N150-treated plants at the same planting density as compared to the N200-treated plants (Figure 1; *p* < 0.05)

**Figure 2:**
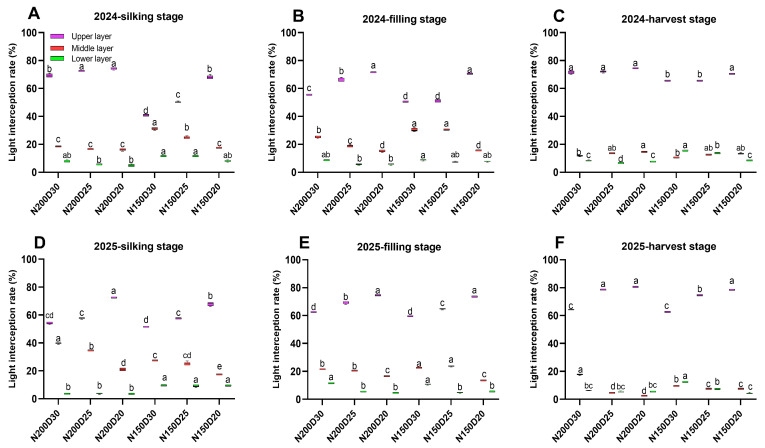
Effect of planting density and nitrogen application rates on light interception rate (IR) across canopy layers during the post-anthesis stage in 2024 and 2025: (**A**–**C**) silking–harvest stages in 2024; (**D**–**F**) silking–harvest stages in 2025. Different lowercase letters indicate significant differences at *p* < 0.05.

The average light interception rate (IR) in the upper canopy was significantly decreased in N150-treated plants from silking to harvest stages (Figure 2; *p* < 0.05) and statistically increased as planting density increased (Figure 2; *p* < 0.05). In contrast, the average IRs in the middle and lower canopy layers were significantly increased in N150-treat plants as compared to N200-treated plants (Figure 2; *p* < 0.05).

### 2.2. Ear Leaf Photosynthetic Characteristics

**Figure 3:**
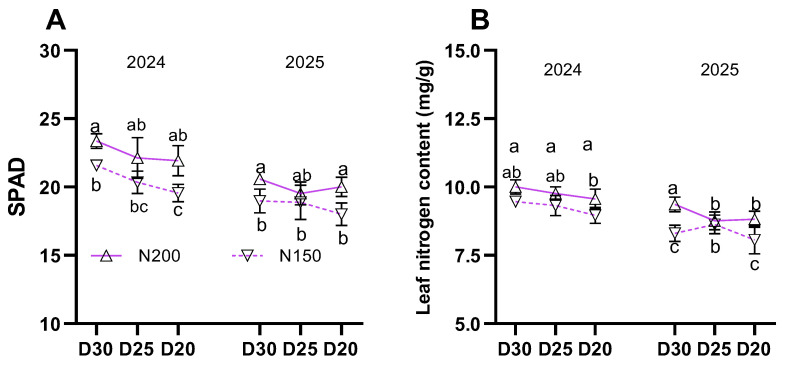
Effect of planting density and nitrogen application rates on (**A**) SPAD value and (**B**) nitrogen content of ear leaf. Different lowercase letters indicate significant differences at *p* < 0.05. Error bars represent SD.

During the filling stage, SPAD values (indicative of chlorophyll content) and nitrogen content in the ear leaf significantly declined with increasing planting density in both years (Figure 3; *p* < 0.05). Plants treated with N200 showed higher average values for these parameters compared to those treated with N150 (Figure 3; *p* < 0.05).

### 2.3. Ear Leaf Gas Exchange

**Figure 4:**
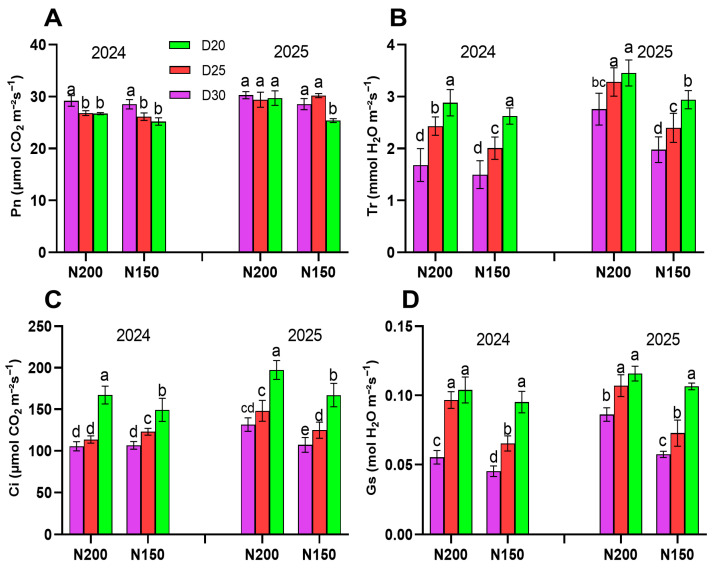
Effect of planting density and nitrogen application rates on gas exchange parameters during filling stage: (**A**) Pn (photosynthetic rate), (**B**) Tr (transpiration rate), (**C**) Ci (intercellular CO_2_ concentration), and (**D**) Gs (stomatal conductance). Different lowercase letters indicate significant differences at *p* < 0.05. Error bars represent SD.

The photosynthetic rate (Pn) decreased with increasing planting density. However, no significant differences were observed between nitrogen levels at the same planting density, except for D20 plants in 2025 (Figure 4A; *p* < 0.05). On average, Pn was 3.57% higher in N200-treated plants compared to N150 in 2024 (27.57 vs. 26.62), and 6.28% higher in 2025 (29.80 vs. 28.04) (*p* < 0.05). Transpiration rate (Tr), intercellular CO_2_ concentration (Ci), and stomatal conductance (Gs) significantly increased with higher planting densities, and their average values were also higher in N200-treated plants compared to those treated with N150 (Figure 4B–D; *p* < 0.05).

### 2.4. Stem Node Characteristics and Mechanical Strength

**Figure 5:**
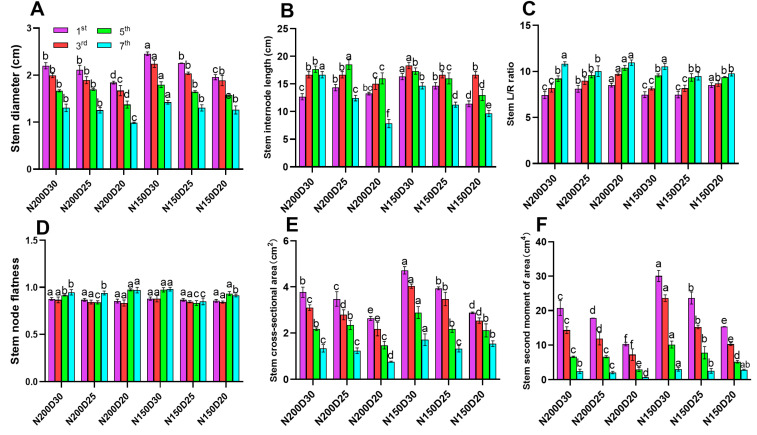
Effect of planting density and nitrogen application rates on stem-node morphological traits: (**A**) diameter, (**B**) node length, (**C**) length-to-diameter ratio, (**D**) node flatness, (**E**) cross-sectional area, (**F**) second moment of area. 1st, 3rd, 5th, and 7th signify the first, third, fifth, and seventh stem node, respectively. Different lowercase letters indicate significant differences at *p* < 0.05. Error bars represent SD.

Stem node diameter, cross-sectional area, and second moment of area decreased significantly with increasing planting density. At the same density, N150 plants exhibited significantly higher values than N200 plants (Figure 5A,E,F; *p* < 0.05). Stem LDR and node flatness increased with planting density, peaking at the seventh node (Figure 5C,D). Stem length decreased with the increasing of planting density, with maximum length observed at the fifth node in N200 and 3rd node in N150 plants (Figure 5B; *p* < 0.05).

**Figure 6:**
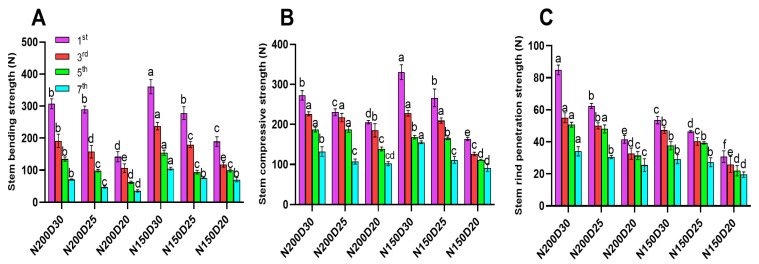
Effect of planting density and nitrogen application rates on stem node mechanical strength: (**A**) bending strength, (**B**) compressive strength, (**C**) rind penetration strength. Different lowercase letters indicate significant differences at *p* < 0.05. Error bars represent SD.

Mechanical strength parameters (bending, compressive, and rind penetration strength) declined as planting density increased (Figure 6; *p* < 0.05). Notably, the average stem bending strength and stem compressive strength were significantly higher in N150-treated plants compared to those treated with N200 (Figure 6A,B; *p* < 0.05). In contrast, rind penetration strength decreased with increasing planting density under the same nitrogen level, and significantly higher values were observed in N200-treated plants (Figure 6C).

### 2.5. Root System Architecture

**Figure 7:**
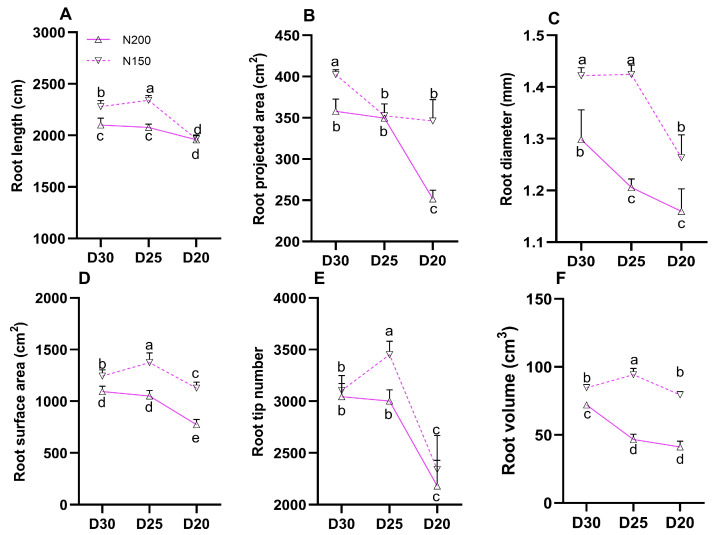
Effect of planting density and nitrogen application rates on root architecture during the 2025 silking stage: (**A**) length, (**B**) projected area, (**C**) diameter, (**D**) surface area, (**E**) tip number, (**F**) volume. Different lowercase letters indicate significant differences at *p* < 0.05. Error bars represent SD.

Root traits, including length, projected area, diameter, surface area, tip number, and volume, were significantly higher in N150 plants at the same planting density. the average increases were 7.24%, 14.13%, 12.29%, 28.29%, 813%, and 61.59%, respectively (Figure 7; *p* < 0.05). The maximum values for root length, root diameter, number of root tips, and root volume were observed under the N150D25 treatment.

### 2.6. Lodging Rate

**Figure 8:**
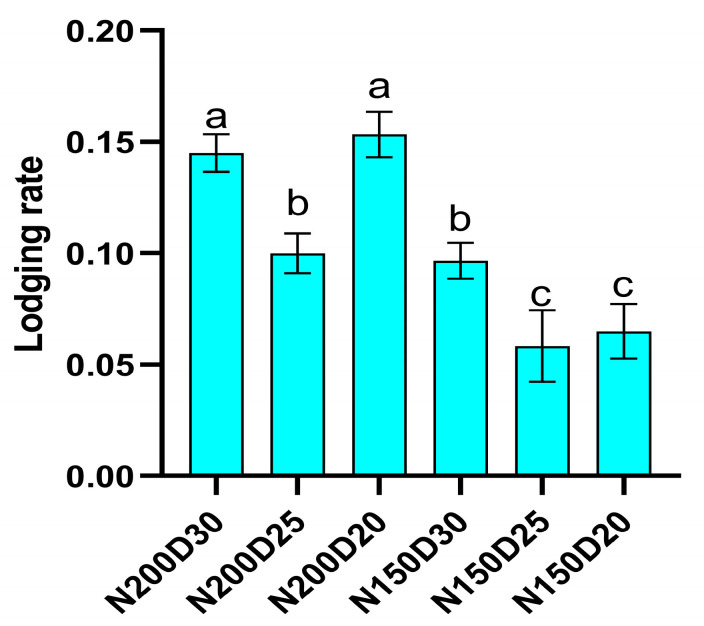
Effect of planting density and nitrogen application rates on sweet corn lodging rate during the 2025 silking stage. Different lowercase letters indicate significant differences at *p* < 0.05. Error bars represent SD.

Root lodging occurred only in 2025 during the filling stage (Figure 8). The average lodging rate was significantly higher under the N200 treatment compared to the N150 treatment. N150D25 plants exhibited the lowest lodging rate (5.83%; *p* < 0.05), while N200 treatment increased lodging by 80.82% on average compared to N150 (13.21% vs. 7.32%; *p* < 0.05).

### 2.7. Ear Commercial Characteristics and Yield

**Figure 9:**
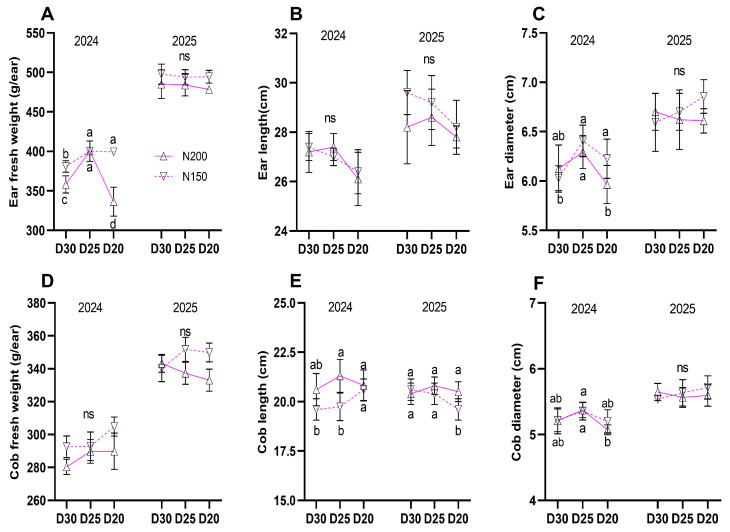
Effect of planting density and nitrogen application rates on commercial ear traits: (**A**) ear fresh weight, (**B**) net fresh weight, (**C**) ear length, (**D**) cob length, (**E**) ear diameter, (**F**) cob diameter. Different lowercase letters indicate significant differences at *p* < 0.05, ns, indicated no significant difference. Error bars represent SD.

Ear fresh weight (except in 2024), cob fresh weight (ear with no husk), and ear length were slightly higher in N150 plants compared to N200 plants, but differences were not statistically significant (Figure 9A,B,D; *p* ≥ 0.05). Cob length was significantly increased under N200 (Figure 9E; *p* < 0.05). However, ear and cob diameters were higher in N150 plants in 2024, while there were no differences observed in 2025 (Figure 9C,F).

The ear yield increased with planting density regardless of nitrogen application level (Table 1; *p* < 0.05). On average, N150-treated plants produced 2.77% (24.79 t/ha vs. 24.12 t/ha) and 3.55% (27.69 t/ha vs. 26.74 t/ha) higher yields than N200-treated plants in the 2024 and 2025 growing seasons, respectively (Table 1; *p* < 0.05). The row number per ear (RNPE) varied between growing seasons. In 2024, RNPE was higher in N200-treated plants (+2.77%; 15.47 vs. 15.06; *p* < 0.05), whereas in 2025, a higher RNPE was observed in N150-treated plants (+7.67%; 18.53 vs. 17.20; *p* < 0.05). However, no significant differences were found in kernel number per row (KNPR) among treatments.

### 2.8. Correlation Analysis

**Figure 10:**
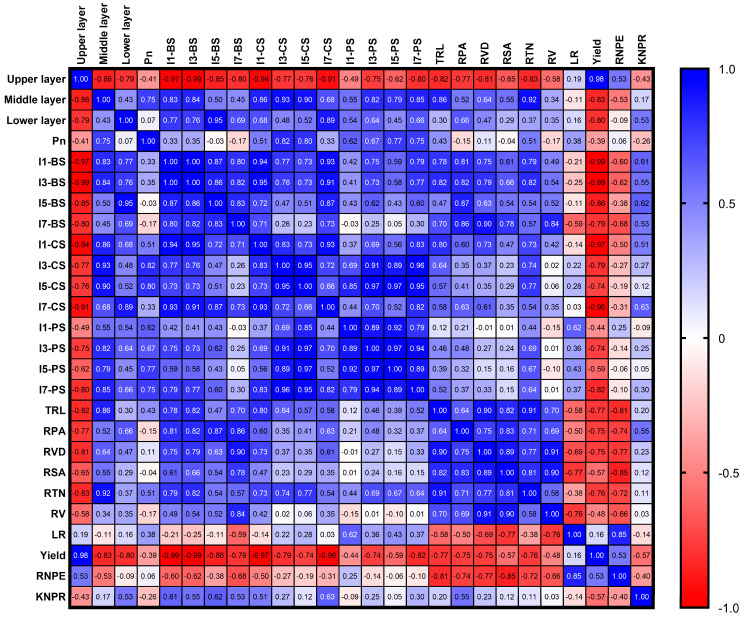
Correlation analysis among light distribution, stem node mechanical properties, root development traits, yield, and lodging rate. The upper layer, middle layer, lower layer, and Pn indicate the light interception rates through the upper, middle, and lower canopy layers and the net photosynthetic rate of the ear leaf, respectively. I1, I3, I5, I7 represent the 1st, 3rd, 5th, and 7th stem nodes, respectively. *BS*—bending strength; *CS*—compressive strength; *PS*—puncture strength; *TRL*—total root length; *RPA*—root projected area; *RVD*—root average diameter; *RSA*—root surface area; *RTN*—root tip number; *RV*—root volume; *LR*—lodging rate; *Yield*—ear fresh yield; *RNPE*—row number per ear; *KNPR*—kernel number per row. Positive and negative correlations are indicated by blue and red cells, respectively. Numerical values within cells represent correlation coefficient values (r).

The correlation analysis revealed distinct relationships between light interception, stem strength, root development, and yield components (Figure 10). The light interception rate through the upper and canopy layers was significantly negatively correlated to the stem node and root system development, but closely positively correlated to ear yield (r = 0.98). The light interception rate through the middle and lower canopy layers, however, had a statistically positive correlation with stem node and root system development and ear leaf Pn (r = 0.75 for the middle canopy layer). Notably, the bending strength of the stem node (BS) and root system development were significantly negatively correlated with the ear yield and lodging rate (LR) (Figure 10).

## 3. Discussion

### 3.1. Effect of Nitrogen Management and Planting Density on Sweet Corn Lodging

Plant lodging, which can occur during the crop life cycle, has been a significant threat to yield formation and improvement around the world. Nitrogen management and planting density are critical agronomic practices for enhancing crop yield. Nitrogen plays an important role in the growth and development of plants and is an important factor in ensuring crop growth and increasing yield and quality. Previous studies indicated that sweet corn is a heavy-fertilizer-requiring crop that requires continuous nutrition throughout its life, especially nitrogen fertilizer, for optimal growth [20]. Many studies note that split nitrogen application is a crucial cropping method to increase nitrogen use efficient by reducing nitrogen volatilization, denitrification, or leaching [21]. However, it may also increase the plant lodging (mainly root lodging) risk. In northern China, the planting density of maize can reach up to 75,000–105,000 plants/ha; however, in tropical China, the planting density of sweet corn is rather lower, nearer to 45,000–60,000 plants/ha. This can be attributed to the following factors: soil properties, climate conditions, plant type (flat-type or compact), etc. Hence, optimizing planting density and nitrogen application is essential for achieving both lodging resistance and high crop yield. In the present study, both increased nitrogen application and higher planting density resulted in an increase in root lodging during the grain filling stage in 2025. These findings are consistent with previous results reported for wheat [22,23] and soybean [24].

### 3.2. The Relationship Between Root–Shoot Development and Plant Lodging

Lodging can be categorized into root lodging and stem breakage, both of which frequently occur during the post-anthesis stage in cereal crops such as rice [25], maize [26], and wheat [27]. In sweet corn, lodging resistance is closely associated with root system architecture and basal stem node development [6]. Several studies have demonstrated that root morphological traits such as root length and root volume are negatively correlated with root lodging [28,29]. Stem quality is another key factor in determining lodging resistance. Cell wall components such as cellulose, hemicellulose, and lignin are critical indicators of stem quality [9]. Mechanical properties of the stem, including bending strength, compressive strength, and puncture strength, significantly influence lodging resistance [30]. Previous study reported a significant positive correlation between root anchorage strength and stem bending strength in maize, highlighting the critical role of root–shoot interactions as a potential determinant of lodging resistance [28]. However, Xue et al. found no such association between root and basal stem lodging resistance [31]. Our previous study demonstrated that root–shoot interaction is a key factor influencing lodging in sweet corn. Optimal nitrogen fertilization and planting density significantly increased the root-to-shoot ratio by enhancing nodal root number and root dry weight, thereby improving root lodging resistance and yield potential [6]. These findings are consistent with the conclusions of [28]. In the present study, the stem bending strength and compressive strength of the basal stem nodes decreased with increasing nitrogen fertilizer and planting density, with the highest average values observed in plants treated with N150D30. Moreover, the root system architecture, characterized by parameters such as root length, root volume, and root diameter, was more developed in N150D25 (expected root projected area). Therefore, the application of N150D25 enhanced root-lodging resistance in sweet corn during the filling stage by balancing root–shoot development. These results indicated that a reduction of 25% of nitrogen input can improve sweet corn lodging resistance by balancing root–shoot development, which is consistent with our previous study [6].

### 3.3. The Relationship Among Plant Architecture, Root–Shoot Development, and Lodging

Plant architecture plays a crucial role in regulating light distribution throughout the canopy. Tian et al. proposed the “Smart architecture” concept, where a narrower upper canopy and an expanded lower canopy enhance light utilization efficiency by improving light penetration to lower leaves [32]. The light distribution through canopy layers was closely associated with stem, root, and sink development [33]. Improving light interception in the lower canopy has been shown to facilitate basal stem node quality and root development, thereby improving lodging resistance [13,34]. The canopy micro-environment and leaf traits play a critical role in determining photosynthetic capacity [35,36]. During the filling stage of maize, ear leaves serve as the primary source of photo-assimilates for ear development [37]; however, this significantly decreased the stalk filling degree and lodging resistance. Therefore, delayed senescence of ear leaves and lower leaves is considered an effective strategy for achieving high yield and lodging resistance in maize production during the post-anthesis stage [38,39].

In the present study, light interception in the upper canopy layer was significantly higher in N200-treated plants during the post-anthesis period, while light interception in the middle and lower canopy layers was higher in N150-treated plants during the post-anthesis stage. This redistribution of light contributed to enhanced Pn during the filling stage, as well as improved basal stem node strength and root system development. Pearson correlation analysis revealed that light interception in the upper canopy layer correlated positively with yield but negatively with basal stem node strength. In contrast, light interception in the middle and lower canopy layers showed positive correlations with both the basal stem nodes development and root system architecture. These results are consistent with the findings of Xue et al. [16] and Xue et al. [31].

### 3.4. Yield and Yield Component

In the present study, we noticed that the Pn was negatively correlated with the yield (r = −0.39, weak correlation), this phenomenon can be attributed to the following reasons: (1) the Pn of ear leaf represents the individual yield of single plant, not field level (population); (2) the photosynthetic assimilation from ear leaf not only allocated to the sink (ear), but also distributed to other vegetative organs like stem and root system; therefore, the allocation and accumulation of dry matter (photosynthetic assimilation) to ear are significant factor to determine final yield. Previous studies demonstrated that an increase in planting density is an efficient agronomic method to increase ear yield, while simultaneously increasing the risk of lodging, which causes yield loss [6]. Optimal nitrogen management can compensate for yield loss caused by plant lodging under dense planting. The number of kernel rows per ear (RNPE) and the number of kernels per row (KNPR) of an individual plant are the most influential factors for ear yield. The former factor can be determined during spike formation at the bell top stage, while the latter can be calculated in the filling stage. Our previous study indicated that reducing nitrogen application and increasing planting density can upregulate RNPE formation, while keeping KNPR limited and without yield loss [6]. This result indicated that RNPE is the strongest contributing factor to determining final yield in sweet corn produce. In the present study, there was no difference observed in KNPR among the treatments, while the average RNPE and yield were significantly higher in N150-treated plants as compared to those in N200-treated plants. Appearance quality is an important factor in determining commercial property. In the present study, the ear fresh weight, ear length, ear diameter, and cob fresh weight were statistically higher in N150-treated plants than in N200-treated plants. These results indicated that reducing the application of nitrogen nutrients can increase commercial property by shaping good appearance quality, which was consistent with our previous studies [6].

## 4. Materials and Methods

### 4.1. Experimental Site

**Figure 11:**
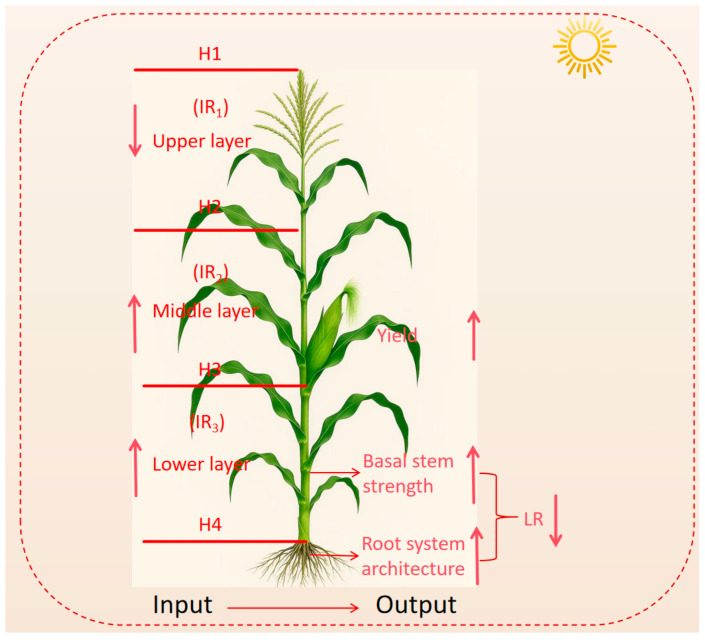
Schematic diagram illustrating the effects of nitrogen fertilization on light interception, photosynthetic capacity, and their relationships with yield and lodging resistance in sweet corn. *IR*—light interception rate; H—vertical canopy height; LR—lodging rate. Upward arrows and downward arrows indicate a positive effect and a negative effect, respectively.

A two-year field experiment was conducted at the Research Base of the Institute of Nanfan & Seed Industry, Guangdong Academy of Science, Yazhou District, Sanya, Hainan Province, China (18°21′30″ N and 109°9′54″ E). The experimental site has a tropical marine monsoon climate, with climatic data for the two growing seasons provided in Figure 11. The soil at the site is classified as light loam, with a bulk density of 1.25 g/cm^3^ and silt content of 20.7%. The physical and chemical properties of the 0–30 cm tillage layer are detailed in Table 2.

### 4.2. Experimental Design

The experiment was conducted in two rounds: the first round (2024 season) was sown on 25 December 2023 and harvested on 20 March 2024, the second round (2025 season) was planted on 26 November 2024 and harvested on 19 February 2025. A split-plot design was adopted with two main factors. Nitrogen application served as the main plot factor with two levels: 200 kg ha^–1^ (N200), representing the standard local high-yield nitrogen dose, and 150 kg ha^–1^ (N150), representing a 25% reduction. Planting density was the sub-plot factor with three levels: 62,500 plants ha^–1^ (20 cm spacing, D20), 50,000 plants ha^–1^ (25 cm spacing, D25), and 41,666 plants ha^–1^ (local practice; 30 cm spacing, D30), with a consistent row spacing of 80 cm across all treatments. Before sowing, phosphorus and potassium fertilizers were applied at rates of 75 kg ha^–1^ (P) and 100 kg ha^–1^ (K), respectively. Each treatment was applied three times, and the area of each plot was 30 m^2^. All fertilizations were applied once at pre-sowing. Fertilizer resources included urea (46% N; SINOPEC, Co., Ltd., Beijing, China), calcium superphosphate (16% P_2_O_5_; SDIC Xinjiang Lop Nur Potassium Salt Co., Ltd., Xinjiang, China), and potassium sulfate (52% K_2_O; Guangdong Zhanhua Group Co., Ltd., Zhaoqing, China). Pest, weed, and irrigation management followed standard local practices.

In this study, we used the sweet corn cultivar Jinbaitian 15, developed by Qingdao Jinmama Agricultural Technology Co., Ltd., Qingdao, Shandong Province, China. This variety, known for its tolerance to abiotic stress, is widely cultivated in tropical and subtropical regions of China and is typically planted at a density of 45,000–60,000 plants ha^–1^ in medium-fertility tropical soils. We germinated the seeds in 105-well plug trays filled with nutrient-rich substrate and transplanted the seedlings to the field at the three-leaf stage.

### 4.3. Measurement Procedures

#### 4.3.1. Light Distribution

**Figure 12:**
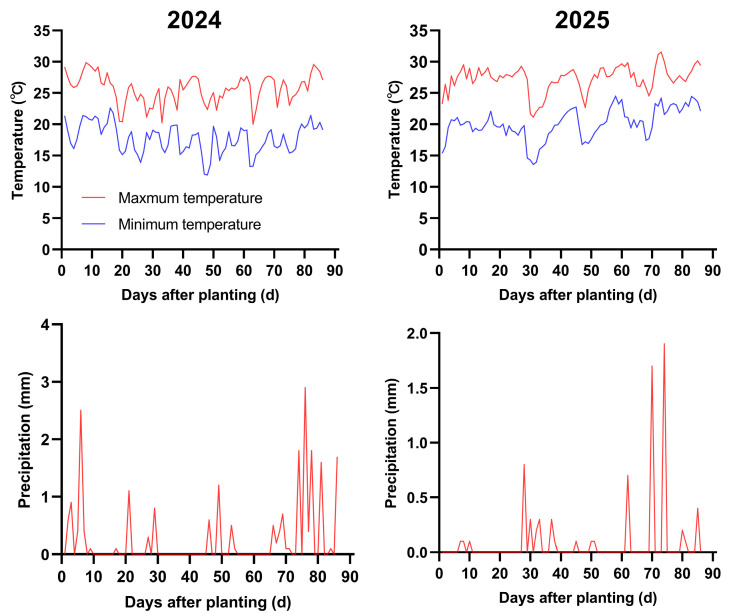
Climate data during both growth seasons.

Light distribution was measured at silking, filling, and harvest stages. The group canopy of each treatment was divided into three layers with four equal vertical height points, namely H1, H2, H3, and H4. The intercepted photosynthetically active radiation (IPAR) of each vertical point was measured. The canopy was divided into the upper canopy layer (vertical height between H1 and H2), middle canopy layer (vertical height between H2 and H3), and lower canopy layer (vertical height between H3 and H4) (Figure 12). The IPAR was measured by using a canopy analyzer (SunScan Canopy Analysis System, Delta-T Devices Ltd., Cambridge, UK) between 10:00 and 11:00 AM on a sunny day. Three independent measurements were taken at each canopy layer for each treatment. The interception rate (IR) for each canopy layer was calculated using the following formula [40]:

IR_n−1_(%) = [(IPARH_n−1_ − IPARH_n_)/IPARH_1_] × 100; n = 1,2,3,4 (1)

#### 4.3.2. Ear Leaf Photosynthetic Capacity

During the grain-filling stage, photosynthetic gas exchange was measured on five replicate plants per plot using an LI-6800 portable photosynthesis system equipped with an LED leaf chamber (Li-Cor Inc., Lincoln, NE, USA). Measurements were conducted on the ear leaf (a representative middle canopy leaf) under standardized conditions: a photosynthetic photon flux density of 1000 μmol photons m^–2^ s^–1^ and a CO_2_ flux of 400 μmol m^–2^ s^–1^ on sunny days. These defined conditions allowed for the estimation of photosynthetic capacity based on steady-state gas exchange parameters. The net photosynthetic rate (Pn), interstitial carbon dioxide content (Ci), stomatal conductance (Gs), and transpiration rate (Tr) were measured and recorded.

#### 4.3.3. Chlorophyll Content and Nitrogen Content in Ear Leaf

Chlorophyll content (SPAD) and leaf nitrogen content were measured between the silking and harvest stages using a TYS-4N handheld chlorophyll meter (Beijing Jinkelida Electronic Technology Co., Ltd., Beijing, China).

#### 4.3.4. Stem Node Characteristics and Mechanical Strength

Stem morphology and mechanical strength were evaluated during the silking stage. Five representative plants per plot were selected, and the length (IL) and diameter (ID) of the first, third, fifth, and seventh internodes were measured using a Vernier caliper, with the first internode being the first one above-ground. Mechanical strength parameters, including bending, compressive, and penetration strength, were measured using a Digital Force Tester (YYD-1, Zhejiang Top Instrument, Hangzhou, Zhejiang, China). The structural traits of stem nodes, specifically internode flatness (IF), length-to-diameter ratio (LDR), cross-sectional area, and second moment of area (SMA), were calculated using the following formulas [41,42]:

IF = a/b(2)

LDR = IL/ID(3)

CSA = (π × a × b)/4(4)

SMA = (π × a^3^ × b)/4 (5)

where a and b represent the outer diameters of the minor and major axes of the stem’s oval cross-section, respectively.

#### 4.3.5. Root System Architecture

Root system architecture was evaluated at the silking stage using the method described by [6]. Root samples from five plants per treatment were excavated, cleaned. The collected whole root of the individual plant was fully expanded with clean water in a transparent plate (20 cm × 30 cm) and scanned via a root system scanner (EPSON EXPRESSION 10000XL, China (Epson Korea Co., Ltd., Seoul, Republic of Korea)). Parameters measured included root length, diameter, volume, tip number, and surface area.

#### 4.3.6. Lodging Rate (LR)

Lodging occurred during the filling stage of the 2025 season (due to strong winds). The lodged plants per plot (LP) and total plants per plot (TP) were calculated, and the lodging rate was calculated as follows:

LR = (LP/TP)(6)

#### 4.3.7. Yield and Yield Components

To assess yield and yield components, ears were harvested from the central three rows of each plot. The total number of ears and the average fresh weight were recorded. Ears from seven representative plants per plot were selected to measure commercial traits, including ear length, ear diameter, cob length, cob diameter, number of kernel rows per ear, and number of kernels per row. Note: cob is taken to mean an ear with no bract.

### 4.4. Statistical Analysis

Statistical analyses were conducted using SPSS 19.0. Treatment differences were evaluated using Duncan’s multiple range test at a significance level of *p* < 0.05. All data are presented as means ± standard error (SE). Figures were generated using GraphPad Prism 8.1.4 for Windows.

## 5. Conclusions

In this study, we believe that reducing nitrogen application and increasing planting density can balance the sweet corn root–shoot development to improve lodging resistance by shaping a reasonable canopy structure (increasing the IR in middle and lower canopy layers). Increased RNPE was the strongest contributing factor to increasing sweet corn yield. However, the plant type (compact vs. flat) of sweet corn, soil properties, and climatic differences should be accounted for in our further studies to confirm the present findings. Collectively, these findings provide a solid foundation for developing high-yield, high-efficiency sweet corn production strategies and scientific guidance for “smart canopy” breeding for sweet corn.

## Figures and Tables

**Table 1:** Effects of nitrogen level (N), planting density (P), and year (Y) on ear yield and yield components.

Years	Treatments	Yield (t/ha)	RNPE	KNPR
2024	N200D30	21.38 d	16.0 a	37.0 a
	N200D25	23.90 c	15.6 a	35.6 a
	N200D20	27.09 a	14.8 c	37.0 a
	N150D30	20.62 e	15.6 a	36.2 a
	N150D25	25.72 b	15.6 a	37.6 a
	N150D20	27.99 a	14.0 d	35.6 a
2025	N200D30	23.57d	16.8 d	40.0 a
	N200D25	26.75 bc	16.8 d	38.0 a
	N200D20	28.91 ab	18.0 b	38.6 a
	N150D30	25.72 c	18.8 a	38.4 a
	N150D25	27.78 b	17.6 c	39.0 a
	N150D20	29.59 a	19.2 a	38.2 a
N		*	*	ns
P		***	ns	ns
Y		**	***	ns
N × P		**	ns	ns
N × Y		**	ns	ns
P × Y		*	*	ns
N × P × Y		***	ns	ns

Note: Data were analyzed using a three-way ANOVA. Different lowercase letters indicate significant differences at *p* < 0.05. *, **, and *** indicate significant differences at *p* < 0.05, *p* < 0.01, and *p* < 0.001. ns—not significant.

**Table 2:** Experimental soil physical and chemical properties.

Growth Seasons	Sand Content (%)	Soil Density (g·cm^−2)^	pH	Organic Matter Content (%)	Alkali Hydrolysable Nitrogen (mg·kg^−1^)	Available Phosphorus (mg·kg^−1^)	Available Potassium (mg·kg^−1^)
2024	49.4	1.41	6.73	1.22	58.56	29.01	73.52
2025	48.0	1.41	6.72	1.18	62.44	36.45	78.66

## Data Availability

Datasets generated during the present study are available from the corresponding author upon reasonable request.
